# Delayed recognition of community transmission of COVID-19 resulting in healthcare worker infections

**DOI:** 10.1017/ice.2020.285

**Published:** 2021-06-10

**Authors:** Raymund B. Dantes, Tait T. Jones, David C. Neujahr

**Affiliations:** Emory University School of Medicine, Atlanta, Georgia

*Letter to the Editor—*We describe a case of delayed COVID-19 diagnosis due to unrecognized community transmission in Atlanta, Georgia, in mid-February 2020. This case resulted in transmission of COVID-19 to 3 of the 4 healthcare workers present during a diagnostic bronchoscopy procedure where only procedural masks were worn.

**Fig. 1.**
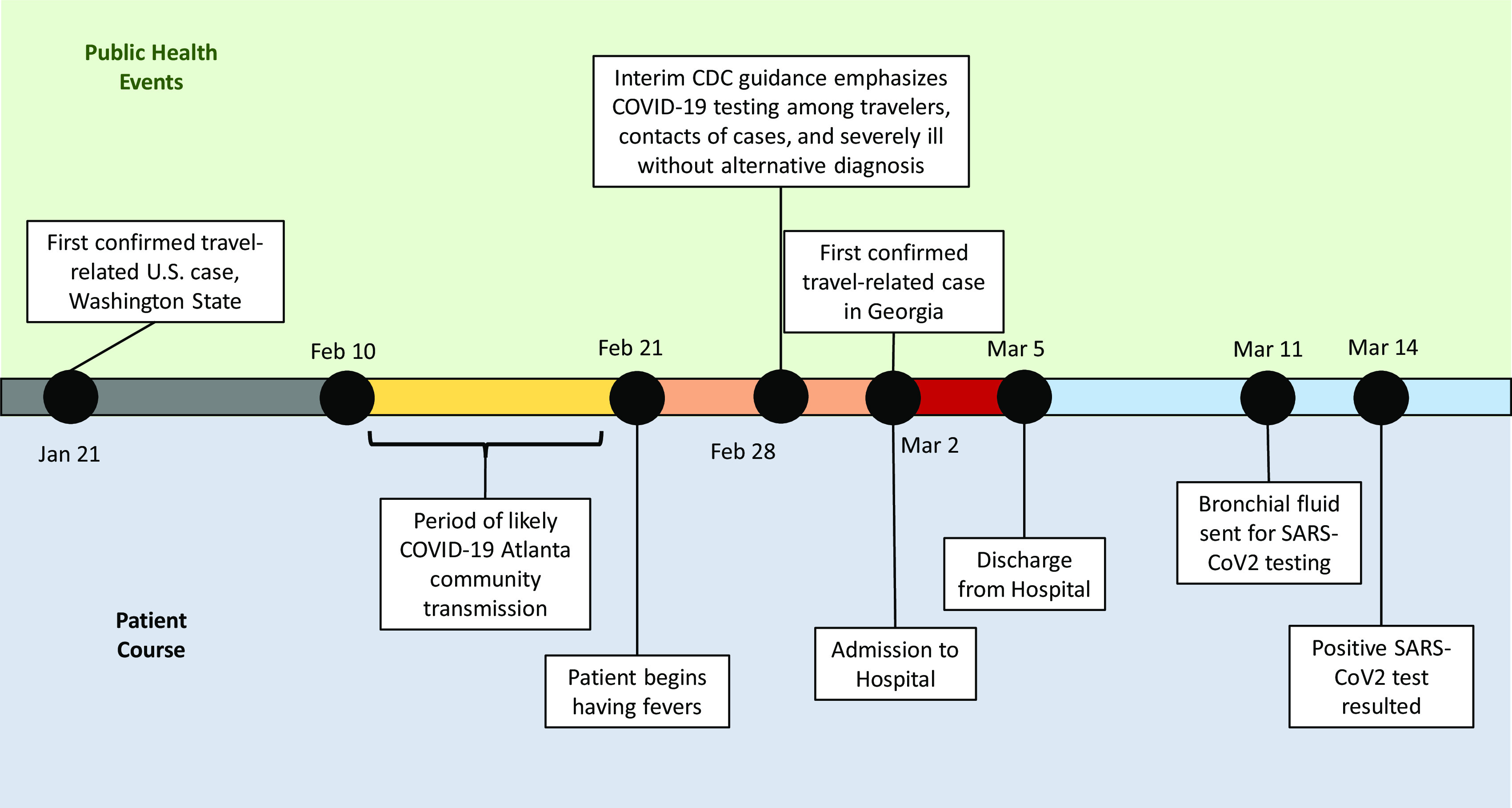
Timeline of public health events and patient course, January 21 through March 14, 2020.

On February 28, 2020 the Centers for Disease Control and Prevention (CDC) announced guidelines recommending COVID-19 testing for persons with compatible symptoms and either recent travel to COVID-19 affected regions, contact with known COVID-19 cases, or “fever with severe acute lower respiratory illness (eg, pneumonia, acute respiratory distress syndrome [ARDS] requiring hospitalization and without an alternative explanatory diagnosis)” (Fig 1).^1^

On March 2, 2020, Georgia officials announced the first 2 cases of coronavirus disease 2019 (COVID-19) in the state, among a Fulton County man who had recently returned from Italy, and his teenaged son.^2^ On the same date, a man aged in his 40s without any known comorbidities presented to the emergency department (ED) for the evaluation of persistent fevers, which began around February 21, 2020. His only other symptom was very mild dyspnea on exertion. He denied any travel outside of the Atlanta area over the past several years. A family member at home had a febrile illness and was recovering at home. He also had a coworker with a febrile illness, but neither contact had any recent travel history. The patient worked in a nearby restaurant frequented by CDC and Emory University employees during lunch breaks, and he resided in Atlanta, Georgia.

His initial evaluation in the ED showed normal vital signs and flu and respiratory syncytial virus rapid tests were negative. He was admitted to the medical floor for further evaluation, where droplet and contact precautions were continued for the remainder of hospitalization. Subsequent evaluation revealed a negative HIV test and negative respiratory viral panel. A computerized tomography scan of the chest revealed “peripheral ground-glass with associated consolidation worse in bilateral lower lobes.” Severe acute respiratory coronavirus virus 2 (SARS-CoV-2) testing was considered and discussed among the hospital medicine, infectious disease, and pulmonary consultation services; however, the patient did not meet the recommended CDC criteria for novel coronavirus testing at that time because he had no relevant travel history, contact with known cases, or severe illness. No commercial or other testing options were available at the time.

On the third day of his hospitalization, he underwent diagnostic bronchoscopy, and staff wore procedural masks. The patient was discharged later that day because he was feeling well and his fever had resolved. He was instructed to remain isolated at home for at least 1 week. Bronchoalveolar lavage fluid was sent for bacterial, fungal, and AFB cultures, which were negative. BAL fluid cellular differential was notable for having 94% macrophages. Cytology was negative for microorganisms, and a Biofire FilmArray Respiratory panel was negative. On March 11, as more SARS-CoV-2 testing capacity became available, his remaining bronchial fluid was mailed to Associated Regional and University Pathologists (ARUP), and it tested positive for SARS-CoV2 virus on reverse transcriptase PCR testing on March 14.

The patient was notified of his test results by telephone, and he reported feeling well with no reoccurrence of fevers or other new symptoms. Within the following week, 3 of the 4 healthcare workers present during the bronchoscopy tested positive for SARS-CoV-2.

This case provides evidence of community transmission of SARS-CoV-2 in Atlanta, Georgia, likely between February 10 and February 19, 2020, based on our current knowledge of the incubation period for SARS-CoV-2.^3^ The precise source of this patient’s COVID-19 remains unknown, but it may have been acquired from either his coworker or a family contact. Due to both restrictive CDC testing criteria and a lack of available SARS-CoV-2 testing outside of public health laboratories, this patient was not diagnosed when rapid public health actions, including contact tracing and isolation, could have limited community spread of this disease. This case also illustrates the risk of SARS-CoV-2 transmission to healthcare workers during bronchoscopy when COVID-19 is not recognized and procedural masks are used instead of N95 or other high-level respirators.
